# In-Hospital Mortality and Hemorrhagic Risks in Traumatic Brain Injury Patients with Early vs. Late Venous Thromboembolism

**DOI:** 10.1055/a-2616-1673

**Published:** 2025-06-18

**Authors:** Sophie Samuel, Jalon Barnes, Lynn Yamane, Eugene Uh, Cyprian C. Afunugo, Bosco Seong Kyu Yang, Huimahn Alex Choi

**Affiliations:** 1 Memorial Hermann Texas Medical Center, Houston, Texas, United States; 2 University of Texas, McGovern Medical School at UT Health, Houston, Texas, United States

**Keywords:** traumatic brain injury, venous thromboembolism, anticoagulation, subdural hematoma

## Abstract

**Objective:**

This study reviewed the management and outcomes of traumatic brain injury (TBI) patients who developed venous thromboembolism (VTE) during hospitalization, focusing on the timing of VTE diagnosis and anticoagulation initiation.

**Methods:**

This retrospective, single-center study utilized data from the University of Texas Trauma Database. Patients were categorized based on VTE diagnosis timing (early ≤7 days, late >7 days). The primary outcome was in-hospital mortality. Secondary outcomes included mortality specifically among patients who were receiving anticoagulation treatment, hemorrhagic complications, predictors associated with early anticoagulation initiation (defined as ≤ 7 days from VTE diagnosis), and whether anticoagulation timing influenced mortality.

**Results:**

Among 237 patients (early: 145, late: 92), the mean age was 59 ± 20 years vs. 55 ± 20 years ( *p* = 0.133). Males comprised 68% vs. 78% ( *p* = 0.038). Subdural hematomas were the predominant injury (63% vs. 68%, *p* = 0.443). In-hospital mortality was similar (10% vs. 13%, *p* = 0.524) and did not differ between anticoagulated and non-anticoagulated patients ( *p* = 0.94). Among patients who died, 73% in the early group and 100% in the late group had received anticoagulation ( *p* = 0.053). Hemorrhage expansion was more frequent in early VTE patients (40% vs. 0%, *p* = 0.046). Pulmonary embolism was associated with early anticoagulation (OR = 1.86, 95% CI: 1.09–3.17, *p* = 0.023), while severe neurologic injury (GCS <9) reduced its likelihood (OR = 0.53, 95% CI: 0.28–0.98, *p* = 0.042).

**Conclusion:**

In-hospital mortality did not differ by VTE timing or anticoagulation status. However, hemorrhage expansion was more frequent in early VTE patients, particularly those with subdural hematomas, emphasizing the need for individualized anticoagulation strategies.

## Introduction

Traumatic brain injury (TBI) is a well-established risk factor for venous thromboembolism (VTE), with an estimated incidence of VTE of around 25% in patients with isolated head injury, and even higher in patients with major trauma. 1 2 3 4 5 6 7 8 For patients who develop VTE, such as deep vein thrombosis (DVT) or pulmonary embolism (PE), clinicians face a significant treatment dilemma: initiating anticoagulation early may prevent the worsening of VTE, but it carries the risk of exacerbating hemorrhagic injuries such as subdural hematomas (SDH), which have a high propensity for re-bleeding and rapid clinical deterioration. 9 10 11 12 13 14 15 16 17

In the early days following TBI, particularly when VTE is diagnosed within the first week, clinicians must navigate a challenging clinical equipoise. On one hand, delaying anticoagulation increases the risk of progression of DVT or PE, potentially leading to fatal outcomes; on the other hand, initiating anticoagulation too early risks exacerbating intracranial hemorrhages such as SDH. The timing of anticoagulation is therefore critical, as the window for safely balancing these risks is narrow. Studies have demonstrated that early anticoagulation in TBI patients, especially those with intracranial hemorrhages, can increase the risk of hemorrhagic expansion, while late anticoagulation may result in thromboembolic complications. 13 14 18

SDH is particularly concerning because, unlike some other types of intracranial hemorrhages, it has a well-documented risk of recurrent hemorrhages and expansion long after the initial trauma. 19 Even after the acute period, when the risk of hemorrhagic expansion for most intracranial injuries decreases sharply, traumatic SDH can continue to enlarge and become symptomatic weeks or even months after TBI, with a significant spontaneous re-hemorrhage rate. 10 11 20 This delayed re-bleeding poses a serious challenge in managing anticoagulation in patients with SDH, as the risk of worsening the hematoma remains long after the initial trauma.

Despite the prevalence of this clinical challenge, there remains a lack of clear guidelines on the optimal timing for initiating anticoagulation in TBI patients with concomitant VTE and intracranial hemorrhages. Previous studies, including those by Kia et al, 20 have highlighted the significant re-bleeding risks in patients with residual SDH who resume anticoagulation, with re-bleeding rates reported as high as 41.2%. However, clinicians are often left to balance these risks on a case-by-case basis without robust evidence to guide decision-making, leading to considerable variability in practice.

The primary objective of this study was to evaluate the association between the timing of anticoagulation initiation (early ≤ 7 days vs. late > 7 days after VTE diagnosis) and in-hospital mortality in patients with TBI. The secondary objectives were to assess mortality while on anticoagulation, evaluate hemorrhagic complications, including hemorrhage expansion, identify patient and clinical factors that influence the timing of anticoagulation initiation, and determine whether anticoagulation timing has a direct effect on in-hospital mortality.

## Methods

### Study Design

This retrospective observational study was conducted at Memorial Hermann–Texas Medical Center, a large academic medical center in Houston, Texas.

### Patient Population

Our study included all adults admitted to Memorial Hermann–Texas Medical Center with a TBI between January 1, 2016 and June 30, 2023. Inclusion criteria were adults with acute TBI, defined by a Glasgow Coma Scale (GCS) score of ≤15 and a confirmed presence of intracranial hemorrhage on admission computed tomography (CT) scan, aged 18 years or older, and diagnosed with VTE during their hospital stay. Patients were excluded if they had contraindications such as active bleeding disorders or recent surgeries that precluded the use of direct oral anticoagulants, warfarin, unfractionated heparin, and low-molecular-weight heparin, were pregnant or actively lactating, or required anticoagulation for another indication outside of the study interest. Patients with TBI but without a diagnosis of VTE, or those with TBI without confirmed intracranial hemorrhage, were also excluded from the study.

VTE diagnosis was confirmed using imaging-based criteria to ensure accuracy and consistency. DVT was diagnosed via Doppler ultrasound, assessing venous flow abnormalities in the lower extremities or other affected regions. PE was identified using computed tomography pulmonary angiography (CTPA), the gold-standard imaging modality for detecting pulmonary emboli. These diagnostic approaches align with standard clinical practice and institutional protocols for evaluating VTE in trauma patients.

All patient data were extracted from the University of Texas (UT) Trauma database. The University of Texas (UT) Trauma Database is a prospectively maintained registry that collects detailed clinical, demographic, and outcomes data on trauma patients admitted to Memorial Hermann–Texas Medical Center, a Level 1 trauma center affiliated with the University of Texas Health Science Center at Houston. Data quality is ensured through standardized data entry procedures, routine audits, and validation checks to maintain accuracy and consistency in reporting. This study, titled “In-Hospital Mortality and Hemorrhagic Risks in Traumatic Brain Injury Patients with Early vs. Late Venous Thromboembolism,” received approval from the UT Health Science Center at Houston Institutional Review Board (IRB) on August 29, 2023 (HSC-MH-23-0749). The procedures carried out during this study were conducted in strict accordance with the ethical standards established by the IRB and adhered to the principles outlined in the Helsinki Declaration of 1975.

### Intervention and Comparison

Our study cohorts were divided into two groups based on the timing of VTE diagnosis relative to the time of TBI: early diagnosis (≤7 days) and late diagnosis (>7 days). The definition of early anticoagulation (≤7 days) was selected based on existing literature, clinical practice, and institutional data, with a primary focus on the risk of delayed hemorrhagic expansion in SDH. Unlike other intracranial hemorrhages, SDH has a prolonged risk window for rebleeding and expansion, making anticoagulation timing particularly complex. Although the highest rebleeding risk occurs within the first 72 hours, thromboembolic complications increase significantly beyond 7 to 10 days, necessitating a careful balance. 21 Additionally, data from our institutional trauma registry indicate that anticoagulation initiation in TBI patients with VTE most commonly occurs around day 8, 22 reflecting real-world decision-making. Given these considerations, a 7-day threshold was chosen as a reasonable and clinically relevant definition of early anticoagulation. For both early and late diagnosis groups, we assessed how many patients were started on anticoagulation early (≤7 days from diagnosis), initiated late (>7 days from diagnosis), or not initiated at all. By categorizing patients in this manner, we aimed to identify the factors influencing anticoagulation timing and assess its association with complications, including hemorrhage expansion, as well as mortality trends.

At our institution, the standard practice for VTE prophylaxis in TBI patients is to initiate pharmacologic prophylaxis within 24 to 48 hours of presentation, unless a clear contraindication is present. In cases where anticoagulation is deemed unsafe, prophylaxis is withheld until reassessment determines it to be appropriate. Although detailed timing of initiation was not available, the majority of patients in this study who were eligible received pharmacologic prophylaxis within this timeframe. Data on mechanical prophylaxis use were not captured.

### Outcomes

The primary outcome of this study was in-hospital mortality stratified by VTE diagnosis timing (early ≤7 days vs. late >7 days after TBI). The secondary outcomes included mortality while on anticoagulation, hematoma expansion, including hemorrhage progression documented via CT scans at the time of death or clinical deterioration, predictors of early anticoagulation initiation, and an evaluation of whether anticoagulation timing has a direct effect on in-hospital mortality. Additional secondary outcomes included the lowest GCS score at discharge, hospital length of stay, ICU length of stay, mechanical ventilator use and duration, presence of hospital complications such as infections, thromboembolic events, or hemorrhagic progression, and the need for blood transfusions. A subgroup analysis was performed on patients with SDH to compare mortality and hematoma expansion rates between those receiving early versus late anticoagulation, given the increased risk of hemorrhagic progression in this TBI subtype. Further analysis was conducted to compare mortality rates between patients who received anticoagulation and those who did not to assess whether anticoagulation had a significant impact on mortality.

### Statistical Analysis

Descriptive statistics were used to analyze the general population in the study. For univariate analyses, the Student's t-test was used for normally distributed continuous variables, while the Wilcoxon Rank-Sum test was used for non-normally distributed continuous variables based on Shapiro-Wilk test results. Categorical variables were compared using the Chi-square test when expected frequencies were sufficient and the Fisher's exact test when cell counts were low. A multivariable logistic regression was performed to identify factors associated with the initiation of anticoagulation within 7 days of VTE diagnosis in patients with TBI. The model specifically focused on identifying predictors of early anticoagulation initiation among those diagnosed with VTE during the initial 7-day post-trauma period. All statistical analyses were performed using RStudio (version 2022.12.0.353; RStudio, PBC, Boston, MA), an integrated development environment for R (version 4.1.3 (2022-03-10); R Foundation for Statistical Computing, Vienna, Austria).

## Results

**Fig. 1:**
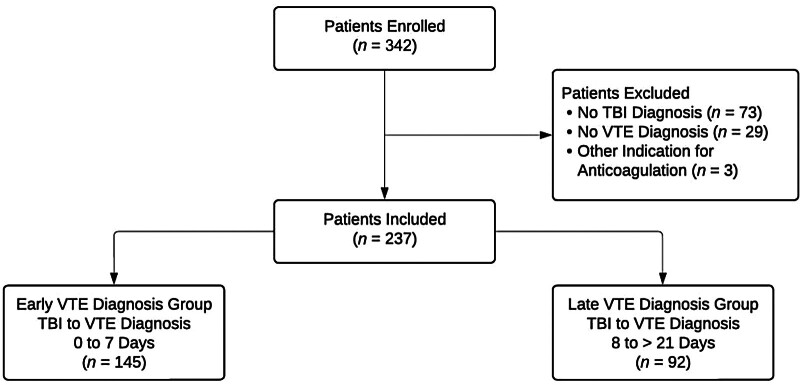
**Patient Flowchart.** The flowchart outlines the patient enrollment process, with a total of 342 patients initially considered. After exclusions, 237 patients were included in the study. These patients were then divided into two groups: the early VTE diagnosis group (diagnosed with VTE within 7 days of TBI, *n* = 145) and the late VTE diagnosis group (diagnosed with VTE between 8 and 21 days after TBI, *n* = 92). TBI, traumatic brain injury; VTE, venous thromboembolism.

The study included 237 patients, with 145 in the early group and 92 in the late group ( Fig. 1 ). Baseline demographics were largely similar, including age, sex, weight, and racial/ethnic distribution, with no statistically significant differences. SDH was the predominant injury type in both groups (64% vs. 67%, *p* = 0.608). However, patients in the late group exhibited more severe neurological impairment, with significantly lower median GCS scores (7 vs. 14, *p* < 0.001), higher rates of midline shift (73% vs. 50%, *p* < 0.001), major extracranial injuries (51% vs. 15%, *p* < 0.001), and non-evacuated hematomas (49% vs. 18%, *p* < 0.001). Anticoagulation initiation was comparable between groups (81% early vs. 88% late, *p* = 0.064), with a median initiation time of 1 day in the early group and 0 days in the late group ( *p* = 0.092). The distribution of DVT and PE was similar across both groups ( Table 1 ).

**Table 1:** Baseline characteristics

Parameter	Early group ( *n* = 145)	Late group ( *n* = 92)	*p* -value
**Demographics**			
Age (mean ± SD)	59.2 ± 20.3	54.7 ± 19.9	0.096
Weight (kg)	82 (70–95)	85 (72–100)	0.255
Male	98 (68%)	70 (76%)	0.160
Hispanic ethnicity (%)	9 (6%)	10 (11%)	0.198
**Race**			
White	84 (60%)	43 (47%)	0.092
Black	22 (15%)	14 (15%)	0.992
Other/Unknown	39 (27%)	35 (38%)	0.071
**Admission characteristics**			
Type of TBI			
SDH	93 (64%)	62 (67%)	0.608
TSAH	37 (26%)	14 (15%)	0.060
Other	15 (10%)	16 (17%)	0.117
Admission SBP (mmHg)	107 (89–122)	105 (83–123)	0.118
Admission GCS (median [IQR])	14 (6–15)	7 (3–14)	<0.001
Lowest admit platelet (10 ^3^ /uL)	163 (129–207)	162 (125–206)	0.765
Lowest admit Hgb (g/dL)	11 (9–13)	11 (10–12)	0.848
**Pupillary response**			
Both reactive (%)	130 (83%)	52 (63%)	0.001
One reactive (%)	8 (5%)	12 (15%)	
Non-reactive (%)	18 (12%)	18 (22%)	
**CT scan findings**			
Major extracranial injury (%)	24 (15%)	42 (51%)	<0.001
Presence of petechial hemorrhages	12 (8%)	28 (34%)	<0.001
Obliteration of ventricles (%)	72 (46%)	50 (61%)	0.030
Subarachnoid bleeding (%)	80 (51%)	62 (76%)	0.002
Midline shift (%)	78 (50%)	60 (73%)	<0.001
Non-evacuated hematoma (%)	28 (18%)	40 (49%)	<0.001
**VTE characteristics**			
Received VTE prophylaxis (%)	132 (91%)	89 (97%)	0.088
**VTE diagnosis type**			
PE	72 (50%)	46 (50%)	0.959
DVT	73 (50%)	46 (50%)	0.959
**TBI to VTE diagnosis time**			
VTE from 0 to 7 days	145 (100%)	–	–
VTE from 8 to 14 days	–	53 (58%)	–
VTE from 15 to 21 days	–	27 (29%)	–
VTE beyond 21 days	–	12 (13%)	–
**Anticoagulation initiation**			
Received AC (%)	117 (81%)	81 (88%)	0.064
No AC received (%)	28 (19%)	11 (12%)	0.064
Time from VTE to AC start (days)	1 (0–2)	0 (0–1)	0.092

Abbreviations: AC, anticoagulation; CT, computed tomography; DVT, deep vein thrombosis; GCS, Glasgow Coma Scale; Hgb, hemoglobin; IQR, interquartile range; PE, pulmonary embolism; SBP, systolic blood pressure; SDH, subdural hematoma; TBI, traumatic brain injury; TSAH, traumatic subarachnoid hemorrhage; VTE, venous thromboembolism.

### Outcomes and Subgroup Analysis

Table 2 summarizes the primary and secondary outcomes for patients in the early and late groups. The primary outcome of in-hospital mortality showed no significant difference between the early group (10%) and the late group (13%) with a *p* -value of 0.524. However, mortality while on anticoagulation trended toward significance, with the early group showing 73% mortality compared to 100% in the late group ( *p* = 0.053). Hemorrhage expansion while on anticoagulation was noted in 40% of patients in the early group (5/15), while no patients in the late group experienced hemorrhage expansion ( *p* = 0.046).

**Table 2:** Primary and secondary outcomes

	Early group *N* = 145	Late group *N* = 92	*P* -value
*Primary outcome*			
Mortality	15 (10)	12 (13)	0.524
Mortality with AC	11/15 (73)	12/12 (100)	0.053
Hemorrhage expansion while on AC as per CT	5/15 (40)	0/12 (0)	0.046
*Secondary outcomes*			
Lowest discharge GCS	14 (12–15)	11 (9–14)	<0.001
Hospital LOS, days	14 (7–26)	26 (18–37)	<0.001
ICU LOS, days	5 (1–16)	17 (8–23)	<0.001
Mechanical ventilator	78 (54)	79 (86)	<0.001
Total ventilator days	1 (0–8)	9 (3–16)	<0.001
Hospital complications a	35 (24)	43 (47)	<0.001
Blood transfusions	58 (40)	42 (46)	0.391

Abbreviations: AC, therapeutic anticoagulation; GCS, Glasgow Coma Scale; ICU, intensive care unit; LOS, length of stay.

Notes: ^a^ Hospital complications: Renal failure, unplanned ICU admission, unplanned intubation, stroke, delirium, cardiopulmonary resuscitation, alcohol withdrawal, ventilator acquired pneumonia, pressure ulcer, gastrointestinal bleeding.

Categorical data reported as count (%); non-continuous data reported as median (IQR).

Secondary outcomes demonstrated that the early group had better clinical results, including a significantly higher lowest discharge GCS (14 vs. 11, *p* < 0.001), shorter hospital length of stay (14 days vs. 26 days, *p* < 0.001), shorter ICU length of stay (5 days vs. 17 days, *p* < 0.001), fewer total ventilator days (1 vs. 9, *p* < 0.001), and fewer hospital complications (24% vs. 47%, *p* < 0.001). There was no significant difference in blood transfusion requirements between the groups (40% vs. 46%, *p* = 0.391).

Given the higher number of patients in the early group receiving anticoagulation treatment within 7 days of TBI, a regression analysis was performed. Predictors for early anticoagulation initiation included lower GCS scores (<9) and PE diagnosis. Specifically, patients with GCS scores <9 were less likely to receive early anticoagulation, with an odds ratio (OR) of 0.53 (95% CI 0.28–0.98; *p* = 0.042), indicating a 47% reduction in the likelihood of early anticoagulation initiation. Conversely, patients diagnosed with PE were more likely to receive early anticoagulation, with an OR of 1.86 (95% CI 1.09–3.17; *p* = 0.023), suggesting an 86% increase in the likelihood of early anticoagulation initiation ( Table 3 ).

**Table 3:** Logistic regression analysis

Predictor	Early AC received (Y) *n* = 104	Early AC not received (N) *n* = 133	OR	95% CI	*p* -value
Age >60	45 (43)	65 (49)	0.68	0.67–3.52	0.285
Male	74 (71)	94 (71)	0.94	0.37–1.25	0.836
GCS <9	34 (33)	60 (45)	0.53	0.28–0.98	0.042
PE	62 (60)	56 (42)	1.86	1.09–3.17	0.023
SDH	62 (60)	93 (70)	0.73	0.41–1.28	0.265
Wt >90 kg	71 (68)	97 (73)	1.38	0.75–2.56	0.305

Abbreviations: AC, anticoagulation; Y, yes ; N, no; OR, odds ratio; CI, confidence interval; GCS, Glasgow Coma Scale; PE, pulmonary embolism; SDH, subdural hematoma; Wt, weight.

Note: This logistic regression analysis identifies factors associated with early anticoagulation initiation (within 7 days) in TBI patients with VTE. Pulmonary embolism (PE) was a significant predictor of early anticoagulation initiation (OR = 1.86, *p* = 0.023), likely reflecting the urgent need to address this life-threatening condition. In contrast, a low Glasgow Coma Scale (GCS <9) was associated with delayed anticoagulation (OR = 0.53, *p* = 0.042), possibly due to increased concerns about bleeding risk in more critically injured patients. Other factors, including age, gender, presence of SDH, and weight, were not significantly associated with early anticoagulation.

In the subgroup analysis focusing on patients with SDH, it was observed that those in the early group were more likely to receive anticoagulation (75% vs. 88%, *p* = 0.038). Mortality rates between the groups were similar, with 15% mortality in the early group compared to 16% in the late group ( *p* = 0.856). Mortality while on anticoagulation was slightly higher in the late group but was not statistically significant (79% vs. 100%, *p* = 0.118). Hemorrhage expansion while on anticoagulation was only observed in the early group, with 40% experiencing expansion compared to none in the late group ( Table 4 ).

**Table 4:** Subdural hematoma subgroup analysis

	Early group *N* = 93	Late group *N* = 62	*p* -value
Anticoagulation initiation			
Received anticoagulation	70 (75)	55 (88)	0.038
No anticoagulation received	23 (25)	7 (12)	0.038
Time from VTE diagnosis to AC start, d	1 (0–2)	0 (0–1)	0.056
AC started within 7 days after TBI	59 (63)	–	–
AC started after 7 days from TBI	14 (15)	52 (84)	<0.001
Outcomes			
Mortality	14 (15)	10 (16)	0.856
Mortality with AC	11/14 (79)	10/10 (100)	0.118

Abbreviation: AC, therapeutic anticoagulation.

Note: Categorical data reported as count (%); non-continuous data reported as median (IQR).

### Additional Analysis of Anticoagulation (AC) versus No AC

In a separate analysis comparing baseline characteristics and imaging findings between patients who received anticoagulation ( *n* = 198) and those who did not ( *n* = 39), significant differences were noted based on admission CT scans. The median GCS score at admission was lower in patients who eventually received anticoagulation, with a median of 10 (IQR: 3–15) compared to 14 (IQR: 10–15) in those who did not receive anticoagulation ( *p* = 0.002). Both pupils were reactive to light in 83.3% of patients who eventually received anticoagulation, compared to 76.9% in those who did not ( *p* = 0.047). Major extracranial injuries were more prevalent in patients who would later receive anticoagulation, with 41.4% compared to 28.2% in the no anticoagulation group ( *p* = 0.032). There was a trend toward a higher presence of petechial hemorrhages in the anticoagulation group (8.6% vs. 5.1%), although this did not reach statistical significance ( *p* = 0.075). Obliteration of the third ventricle was observed at similar rates in both groups, with 50.5% in the anticoagulation group and 48.7% in the no anticoagulation group ( *p* = 0.649). Subarachnoid bleeding was more frequently observed on admission scans in patients who eventually received anticoagulation (60.1%) compared to those who did not (51.3%, *p* = 0.041). Midline shift was also significantly more common in admission scans in the anticoagulation group (38.4% vs. 23.1%, *p* = 0.019). Non-evacuated hematomas were slightly more common in the anticoagulation group, with 42.4% compared to 35.9% in the no anticoagulation group, though this difference was not statistically significant ( *p* = 0.257). Difference in mortality between the two groups was not significant ( *p* = 0.94) ( Table 5 ).

**Table 5:** Analysis of anticoagulation (AC) vs. No AC

Variable ( *n* , %)	AC received (n = 198)	No AC ( *n* = 39)	*p* -value
**GCS (median, IQR)**	10 (3–15)	14 (10–15)	0.002
**Pupils reactive to light**			0.047
- Both	165 (83.3%)	30 (76.9%)	
- One	12 (6.1%)	5 (12.8%)	
- None	21 (10.6%)	4 (10.3%)	
**Major extracranial injury**	82 (41.4%)	11 (28.2%)	0.032
**Presence of petechial hemorrhages**	17 (8.6%)	2 (5.1%)	0.075
**Obliteration of third ventricle**	100 (50.5%)	19 (48.7%)	0.649
**Subarachnoid bleeding**	119 (60.1%)	20 (51.3%)	0.041
**Midline shift**	76 (38.4%)	9 (23.1%)	0.019
**Non-evacuated hematoma**	84 (42.4%)	14 (35.9%)	0.257
**Mortality**	16 (8.08%)	3 (7.69%)	0.94

Abbreviations: AC, therapeutic anticoagulation; GCS, Glasgow Coma Scale.

## Discussion

This study sought to evaluate the relationship between the timing of VTE diagnosis and anticoagulation initiation, as well as their association with hemorrhagic complications, in-hospital mortality, and clinical decision-making in patients with TBI. Patients with VTE diagnosed early (within 7 days of TBI) exhibited higher rates of hemorrhage expansion compared to those diagnosed late (after 7 days), particularly among those with SDH. Despite the increased risk of hemorrhage expansion in the early group, overall in-hospital mortality did not significantly differ between early and late diagnosis groups. However, since hemorrhage expansion occurred only in the early group and some of these patients died, it remains uncertain whether early anticoagulation contributed to their deaths. Anticoagulation was initiated promptly in both groups, with a median of 1 day in the early diagnosis group and 0 days in the late diagnosis group, reflecting rapid clinical decision-making following VTE identification.

Patients with late-diagnosed VTE were clinically more severe at baseline, as evidenced by lower GCS scores, higher rates of midline shift, and extracranial injuries on admission CT scans. Furthermore, VTE diagnoses were significantly lower in this sicker group, suggesting a potential performance bias. Interestingly, mortality trends were similar between early and late diagnosis groups. Although patients diagnosed late were sicker overall, their VTEs were identified later from the time of TBI, potentially explaining the absence of hematoma expansion in this group. This raises the question of whether the lack of mortality differences implies that anticoagulation might not have a direct effect on mortality.

To further evaluate the role of anticoagulation in mortality, we compared outcomes between patients who received anticoagulation and those who did not. Interestingly, anticoagulation did not directly influence overall mortality, with comparable death rates observed between those who received anticoagulation (9.6%) and those who did not (10.3%). This suggests that mortality may be driven more by the underlying severity of TBI rather than by anticoagulation itself. However, the presence of hematoma expansion in the early group highlights a subset of patients at higher risk of complications that warrants further investigation.

To better understand why the anticoagulated group, which presented sicker than the non-anticoagulated group, received anticoagulation, we conducted a regression analysis. This analysis revealed critical factors influencing anticoagulation timing post VTE diagnosis. Patients with PE were significantly more likely to receive early anticoagulation, with an odds ratio (OR) of 1.86, reflecting the urgency of treating life-threatening embolic events. Conversely, patients with severe neurologic injury, characterized by GCS <9, were less likely to receive early anticoagulation (OR 0.53), indicating clinical hesitancy to initiate anticoagulation in those perceived to be at higher risk for hemorrhagic complications. These findings suggest that the decision to initiate anticoagulation is primarily influenced by the clinical context, such as the presence of PE, rather than solely the timing of VTE diagnosis.

Several studies have explored the risks and benefits of anticoagulation in patients with neurosurgical pathologies, though relatively few have focused specifically on TBI. 13 23 24 25 26 27 28 29 A recurring theme in these studies is the delicate balance between the need for anticoagulation to prevent thromboembolic events and the risk of hemorrhagic complications, particularly in patients with SDH.

One study evaluated the safety of interrupting anticoagulation in patients with intracranial hemorrhage and mechanical heart valves, noting that discontinuation of anticoagulation for 1 to 2 weeks in these patients was safe, with no significant expansion of intracranial hemorrhages. However, there was no specific analysis of SDH, and the study did not detail the amount of residual hematoma at the time of anticoagulation resumption. 24

A study of 55 patients with SDH on anticoagulation or antithrombotic therapy found a 9.1% risk of clinically significant hematoma expansion when therapy was initiated within 14 days of admission. Unfortunately, the study did not provide detailed information on the size or status of the SDH at the time of anticoagulation initiation, which limits its applicability in determining when it might be safe to restart anticoagulation. 13

An analysis of 39 patients anticoagulated for mechanical heart valves who developed intracranial hemorrhage was conducted by Wijdicks et al. Among these patients, 20 had acute SDH. The study found that aggressive reversal of anticoagulation was effective in preventing further hemorrhagic expansion, and no thromboembolic events were reported during the period of anticoagulation interruption. Although this study offered valuable insights into the safety of temporary anticoagulation cessation, it did not specifically evaluate SDH patients in detail, nor did it provide information on residual hematomas at the time of anticoagulation resumption. 26 Byrnes et al investigated anticoagulation in 42 TBI patients with post-traumatic thromboembolic events. In their cohort, 26 patients were anticoagulated, and none of the 13 patients with SDH experienced hematoma expansion after anticoagulation was initiated. However, the study did not detail the size or evolution of the SDH, leaving questions about the safety of anticoagulation in this subset of patients. Moreover, although thromboembolic events were tracked, this study, like others, did not include detailed imaging follow-up to assess the status of SDH at anticoagulation resumption. 23

This study has several limitations inherent to its retrospective design. The lack of detailed imaging follow-up to confirm the resolution of SDH restricts our ability to determine the optimal timing for anticoagulation initiation with minimal hemorrhagic risk. Additionally, we did not differentiate between subtypes of SDH, such as acute or acute-on-chronic, which may have influenced the timing of anticoagulation and the associated risk of re-hemorrhage. Although thromboembolic events were assessed, the absence of comprehensive data on worsening thromboembolic complications during anticoagulation delays and the specific causes of mortality (VTE-related vs. hemorrhage expansion) limits the breadth of our findings. Although hemorrhage expansion was observed exclusively in the early group, and some of these patients died, we were unable to determine whether their deaths were directly attributed to hemorrhage progression. Future studies should incorporate cause-specific mortality assessments and longitudinal imaging data to better characterize the risks associated with anticoagulation timing in TBI patients. Furthermore, the study's single-center nature may limit the generalizability of the results to other institutions or patient populations. The potential for unmeasured confounders, including variations in clinical practice, imaging interpretation, and patient-specific factors, reflects the inherent challenges of retrospective analyses.

Additionally, although this study focused on TBI patients with VTE, we did not restrict inclusion to isolated TBI cases. Some patients had concurrent extracranial injuries, as reflected in Table 1 , but polytrauma data were not systematically collected. This represents a potential confounder, as extracranial injuries could have influenced anticoagulation timing and outcomes. Future studies should explore the impact of polytrauma on clinical decision-making regarding VTE management in TBI patients.

Although no significant difference in in-hospital mortality was observed between early and late VTE groups or between those receiving early versus late anticoagulation, the late VTE group had a significantly longer hospital stay. Given that these patients were clinically more severe at baseline, this extended LOS may reflect greater injury severity rather than anticoagulation timing effects. We accounted for this by performing multivariable regression analysis and subgroup analyses, but residual confounding remains possible.

Despite these limitations, this study has significant strengths. It is one of the few studies to systematically evaluate anticoagulation timing in a large cohort of patients with SDH and VTE, providing valuable insights into clinical decision-making in this high-risk population. The inclusion of regression analysis allowed us to identify specific factors influencing anticoagulation timing, such as PE diagnosis and severe neurologic injury, shedding light on the drivers of clinical decisions. Furthermore, the study's focus on outcomes such as hemorrhage expansion, in-hospital mortality, and baseline injury severity provides a comprehensive view of the risks and benefits of anticoagulation in TBI patients.

## Conclusion

Our findings suggest that early anticoagulation was associated with hematoma expansion in patients with SDH; however, there was no significant difference in overall in-hospital mortality between those who received early versus late anticoagulation or between those who received anticoagulation and those who did not. Although hospital length of stay differed between groups, potentially reflecting underlying injury severity, our analysis accounted for key confounders, including baseline injury severity and anticoagulation timing. These results emphasize the importance of individualized anticoagulation strategies, guided by detailed imaging follow-up and clinical context, to balance the risks of hemorrhagic and thromboembolic complications. Future studies should incorporate imaging and clinical data to refine guidelines and improve outcomes for TBI patients requiring anticoagulation.
